# The role of detritivory as a feeding tactic in a harsh environment – a case study of weatherfish (*Misgurnus fossilis*)

**DOI:** 10.1038/s41598-019-44911-y

**Published:** 2019-06-11

**Authors:** Kacper Pyrzanowski, Grzegorz Zięba, Małgorzata Dukowska, Carl Smith, Mirosław Przybylski

**Affiliations:** 1 Department of Ecology and Vertebrate Zoology, University of Łódź, Łódź, Poland; 2 Institute of Vertebrate Biology, Academy of Sciences of the Czech Republic, Brno, Czech Republic; 3 School of Biology and Bell-Pettigrew Museum of Natural History, University of St Andrews, St Andrews, UK

**Keywords:** Freshwater ecology, Ichthyology

## Abstract

The weatherfish (*Misgurnus fossilis*) is a species that is tolerant of unfavourable environmental conditions and can survive low dissolved oxygen concentrations and high water temperatures. Although this species occurs across almost the whole of Europe, and is protected in many countries, relatively little is known regarding its ecology. To determine the diet of weatherfish, 120 individuals from an artificial drainage canal in central Poland were collected in two seasons (spring and late summer) with contrasting abiotic condition (oxygen concentration, water temperature and transparency). Analysis of gut fullness showed that weatherfish consumed a greater quantity of food in spring (0.92 ± 0.90) compared with summer (0.20 ± 0.26). Contrary to other cobitid taxa, weatherfish fed actively during daytime in both seasons. An estimate of the importance of each dietary component indicated that the most important food categories were chironomids, copepods, *Asellus aquaticus* and detritus. SIMPER analysis indicated that these four categories together constituted over 65.8% of cumulative dissimilarity in the diet between seasons. Additionally, trophic niche breadth differed significantly between seasons. The study demonstrated that the weatherfish is an opportunistic feeder, consuming large quantities of detritus despite possessing a gut morphology that is atypical of a detritivore. The quantity of detritus in the gut of weatherfish was positively associated with fish total length and varied seasonally, with a greater quantity of detritus in the diet in late summer. These results demonstrate the importance of detritus as a source of energy, particularly during periods of scarcity of alternative prey categories.

## Introduction

In the temperate zone, freshwater ecosystems are characterized by natural variation in environmental conditions resulting, *inter alia*, from climate seasonality^1,2^. In shallow ponds, rivers or canals, naturally variability in environmental parameters can be extreme, sometimes with negative consequences for aquatic fauna and potentially causing population declines or extinction^3,4^. In drainage canals, high water flow and high dissolved oxygen concentrations associated with elevated rainfall and snowmelt are usually observed in spring. In the summer, canals accumulate detritus and become overgrown with vegetation, decreasing dissolved oxygen concentrations^5^. Low oxygen concentrations also result from elevated water temperature, accompanied by an increase in water conductivity and decomposition of organic sediments on the substrate^6,7^. Under these conditions there is often a decrease in macroinvertebrate biomass, mainly as a consequence of the loss of oxygen-sensitive taxa and thereby a scarcity of the food resources for fish^8–10^. Drainage canals are often populated by macroinvertebrate and fish with special adaptations that permit them to survive the sometimes harsh environment^11,12^. Under these conditions fish may switch from one food resource to other, comprising less preferred items, as a result of changes in food availability^13^.

One of the few European fish species that can tolerate conditions in drainage canals is the weatherfish, *Misgurnus fossilis* (L), which is able to survive low oxygen tensions (hypoxia) due to its capacity for cutaneous respiration and ability to perform oxygen uptake via its gut^14^. *M. fossilis* is a small, benthic freshwater cobitid, native to almost all of Europe. It inhabits stagnant freshwaters, such as oxbow lakes and ponds, as well as slow-flowing rivers, canals and drainage ditches that are overgrown with dense vegetation^15^. This fish species usually occurs on a sandy substrate covered with a thick layer of mud and organic matter^16,17^. The weatherfish is believed to be a nocturnal omnivore, feeding chiefly on insect larvae, small crustaceans and molluscs as well as on detritus^15^. Despite its unspecialized habitat and feeding requirements and adaptations to poor water quality, it has declined in many regions^16,18^. As a consequence, the weatherfish was listed under Annex II of the EU Council Directive 92/43/EEC, representing a species of European Community concern^19^ and was subsequently included in numerous governmental Red Lists of endangered and protected fishes throughout Europe^18^, including Poland^20^. In Europe, the weatherfish has been classified as a low concern species (LC)^21^, though its threat level might be considered to be higher due to its low genetic diversity^22^.

In light of the limited understanding of weatherfish life history, the aim of the present study was to assess its feeding activity and diet composition in two seasons characterized by contrasting environmental conditions. Thus, the study examined temporal variation in environmental conditions and food availability between two distinct periods to assess how these changes were reflected in the diet composition and feeding patterns of weatherfish. Three specific questions were addressed: (1) whether there is a diel feeding activity in weatherfish; (2) whether there is a seasonal pattern in diet composition, and (3) whether detritus makes a significant contribution to the diet, and whether this varies seasonally.

## Materials and Methods

### Study site

The study was conducted in the Południowy canal, situated on a tributary of the River Bzura (51° 13′14.86″N, 19°48′03.62″E). The canal is 6.5 km long, with an average slope of 0.41‰. The channel is approximately 3 m wide with an average depth of 0.3 m (upper section) to 0.8 m (outlet). The substrate was dominated by sand covered with a thick layer of organic matter. The entire length of the watercourse was overgrown with submerged and emergent vegetation. The banks were covered with reeds and sedges, which together with isolated trees along both banks gave partial shade to the channel. The canal is part of a drainage network of the Natura 2000 Bzura-Ner glacial valley (PLH100006). This area has recently been recognized as a site of high weatherfish abundance in Poland^23^. Previous data show that weatherfish have been abundant in this water network^24–26^.

### Sample collection and processing

A total of 120 weatherfish (mean total length TL – 124 mm, range 87–205 mm), were collected in August 2014 and May 2015 (56 and 64 individuals, respectively) by electrofishing (EFGI 650, BSE Bretschneider Specialelektronik, Germany). Groups of 14–16 fish were collected at 6-h intervals over a 24-h period; at 06.00, 12.00, 18.00 and 00.00. After capture, fish were immediately euthanized with an overdose of clove oil^27^ and preserved in 4% buffered formaldehyde. The weatherfish is protected in Poland, therefore all procedures were carried out under permission from the Local Ethics Committee (66/ŁB729/2014) and the Regional Directorate of Environmental Protection (WPN-II.6401.268.2014.KW2).

In the laboratory all specimens were measured for total length (TL) to the nearest 1 mm and weighed (W) to the nearest 10 mg. The alimentary tracts of each specimen were removed and measured (AtL) to the nearest 1 mm. Linear regression was used to model the relationship between length of alimentary tract and fish total length. Gut contents were weighed to the nearest 1 mg and stored in glycerine. Food items were subsequently identified to the lowest practical taxon; i.e. to order, family or species and/or genus where possible, under a stereomicroscope (Nikon SMZ1000, Japan) and counted. The total number and estimated weight of each prey type were recorded for each fish specimen.

The fullness coefficient (FC), calculated as the percentage of gut content wet weight and fish weight was used to investigate diel feeding activity of weatherfish. One-way analysis of variance (ANOVA I) with Bonferroni *post-hoc* test^28^ were used to determine whether the gut fullness values were significantly different over a diel cycle.

Prey items were combined by taxon and quantified by the frequency of occurrence (%F) and percentage of biomass (%B)^29^. For each food category the index of importance (IRI) was calculated^30^ and its standardized value (%IRI)^31^ estimated as:

$${{\rm{IRI}}}_{i}=100\times {{\rm{HI}}}_{i}\times {{\rm{HI}}}^{-1},\,{\rm{where}}:{{\rm{HI}}}_{i}= \% {{\rm{F}}}_{i}+ \% {\rm{B}}$$

$$ \% {{\rm{IRI}}}_{i}=100\,{{\rm{IRI}}}_{i}/\Sigma {{\rm{IRI}}}_{i}$$

where IRI_*i*_ is the IRI value for each prey category of prey *i* and ΣIRI_*i*_ is the total IRI for all prey categories.

Differences in weatherfish diet between seasons were analysed using a one-way permutation analysis of similarity (ANOSIM, Bray-Curtis similarity coefficient)^32^. ANOSIM is analogous to an ANOVA procedure, with a non-parametric permutation applied to a rank similarity matrix of samples^32^. In this procedure, the R statistic provides an absolute measure of how groups are separated. Generally, R values lies between 0, when groups are indistinguishable, and +1, when all similarities within groups are less than the similarity between groups^33^. The similarity percentage procedure (SIMPER)^32^ was used to identify which prey taxa were most likely responsible for the patterns detected by ANOSIM. SIMPER provided the average dissimilarities between the fish samples and identified the prey categories that made the greatest contributions to any dissimilarity. All multivariate techniques for analysing diet data were conducted using the PAST v3.15 software^34^. Food niche width of weatherfish in different seasons was calculated as trophic diversity indices, Levin’s (*B*) and Shannon-Wiener’s (*H*′), and their standardized forms (evenness indices), *B*_*a*_ and *J*′, respectively defined as:

$$B=1/{\rm{\Sigma }}{p}_{i}^{2};{H}^{{\rm{^{\prime} }}}=-\,{\rm{\Sigma }}{p}_{i}\,{\rm{l}}{\rm{n}}\,{p}_{i};$$

$${B}_{a}=(B-1)/(S-1);$$

$$J^{\prime} =H^{\prime} /\mathrm{ln}\,S$$

where *p*_*i*_ is the biomass proportion of a given food category in the total biomass of all food categories, and *S* is the number of food categories. For all the indices, average values and their standard errors were obtained using the jack-knife technique^35^.

The correlation between detritus abundance (%DA) in the gut of weatherfish and abundance of the other food categories abundance was examined by the Spearman rank correlation coefficient (rs).

The proportion (arcsin transformed) of detritus (*DA*_*i*_) in the alimentary tract was modelled for individual weatherfish *i* as a function of fish total length (*TL*) and collection season (*season*) using a Gaussian GLM. The model was specified as:

$$D{A}_{i}\sim N({\mu }_{i},{\sigma }^{2})$$

$$E(D{A}_{i})={\mu }_{i}\,{\rm{and}}\,var(D{A}_{i})={\sigma }^{2}$$

$${\mu }_{i}={b}_{1}+{b}_{2}\times T{L}_{i}\times {b}_{3}\times seaso{n}_{i}$$

Prior to analysis a data exploration was undertaken to examine the data for outliers in the response and explanatory variables and for zero inflation in the explanatory variable^36^. The model was fitted using R (version 3.5.2)^37^.

## Results

**Figure 1:**
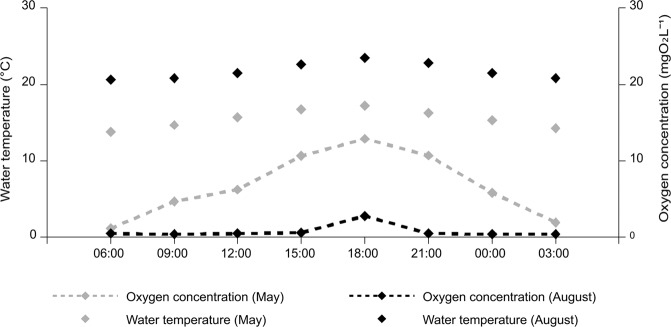
Oxygen concentration mg O_2_ l^−1^ and water temperature (°C) in two seasons (May and August) over a full diel cycle at time intervals between 06.00–03.00 in the Południowy canal.

**Figure 2:**
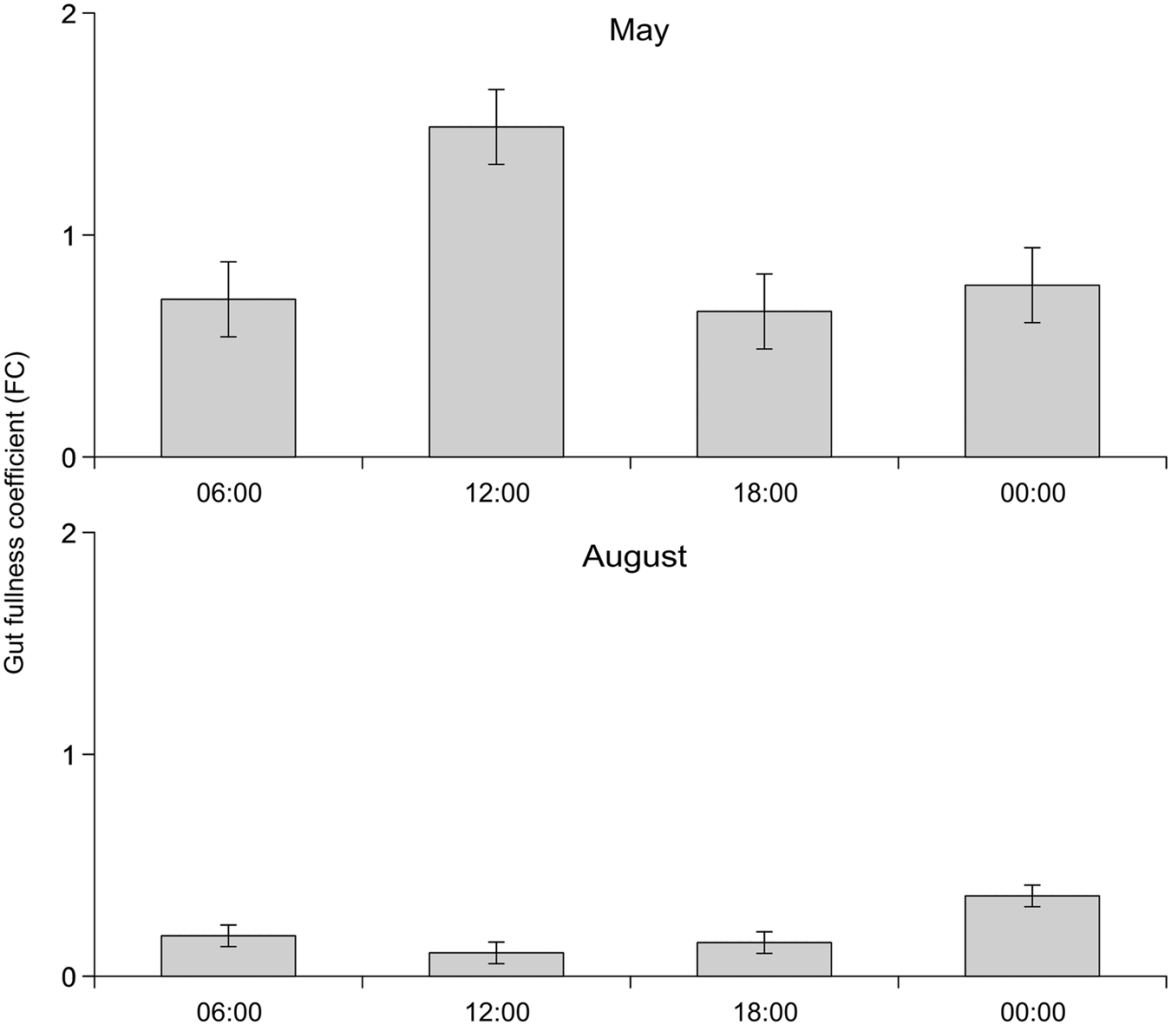
The fullness coefficient (FC) in weatherfish (*Misgurnus fossilis*) in two seasons (May and August) over a full diel cycle at time intervals between 06:00-00:00 in the Południowy canal. Error bars indicate standard error of the mean.

The environmental conditions in the Południowy canal varied seasonally (Table 1). The physical and chemical parameters of the water; i.e. dissolved oxygen and saturation, temperature, conductivity and pH, differed between spring (May) and late summer (August) (Table 1). In particular, dissolved oxygen concentrations in May were over 12 mg l^−1^, while they never exceeded 3 mg l^−1^ in August (Table 1). Moreover, this parameter showed a clear diel pattern of variation with the highest oxygen dissolved concentration observed during daylight hours (with a peak at 18.00) falling to less than 2 mg l^−1^ during the night (Fig. 1). There were also significant differences in the food base, which constitutes the potential prey of weatherfish (PERMANOVA; pseudo F = 4.76, p < 0.001). Weatherfish consumed more food in May, when the average gut fullness coefficient (FC) was 0.92 ± 0.90 (mean ± SD), compared with August (0.20 ± 0.26). In both seasons fish showed a significant diel pattern in feeding activity (May: F_3,60_ = 3.33, p = 0.025; August: F_3,52_ = 2.84, p = 0.047). In May the highest FC values were observed at noon and differed significantly from the values in the afternoon (i.e. 18.00). There was no difference in FC values at 06.00 and midnight (Bonferroni *post-hoc* test, p < 0.05) (Fig. 2). In contrast with the pattern in May, multiple comparisons of FC in August failed to show any significant diel pattern of feeding (Fig. 2). In August a high proportion of fish were found to have empty alimentary tracts; of the 120 weatherfish examined, 27 had an empty gut. Specimens with empty alimentary tracts were recorded primarily during daylight (14 fish), but also during darkness (7). In May, only 5 individuals with an empty gut were recorded and all at night. The proportion of fish with an empty gut was lower in May (*f*_M_ = 0.078) than August (*f*_A_ = 0.375) (p = 0.033).

**Table 1:** Habitat characteristics of the Południowy canal during two seasons (May and August).

Trait	Month	Mean	Min	Max	SD	Mann – Whitney test	
						Z	p
Saturation (O_2_%)	May	69.2	10.9	134.3	45.151	3.360	0.00078
	August	>0.1	>0.1	0.23	0.066		
Oxygen concentration (mgO_2_ l^−1^)	May	6.8	1.14	12.9	4.285	3.116	0.00183
	August	0.8	0.4	2.8	0.826		
Water temperature (°C)	May	15.5	13.8	17.2	1.196	−3.313	0.00092
	August	21.7	20.6	23.5	1.078		
Conductivity (mS cm^−1^)	May	1085.1	1066.0	1103.0	12.495	3.313	0.00092
	August	848.9	841.0	855.0	5.357		
pH	May	7.6	7.5	7.8	0.125	3.311	0.00093
	August	7.1	6.8	7.2	0.114

The analysis of alimentary tract contents showed that in May, among 22 food categories, weatherfish fed primarily on chironomids, copepods, *Asellus aquaticus* and detritus, the latter contributing 10% of diet content with a frequency of occurrence over 39% (Table 2). Estimates of IRI values also indicated that detritus was the most important dietary component for weatherfish (Table 2). However, other constituents of the diet; such as ostracods, chydorids, beetle larvae, gastropods and plant material, were also consumed frequently (29%), but with lower abundance (from 3.4 to 5.0% in diet composition). The other 12 food categories identified in the diet can be considered as unimportant food resources (Table 2). In August the diet composition was much more restricted and chironomids and detritus were the main food sources for weatherfish. Although both these food categories showed relatively high IRI values, they were lower than in May (Table 2).

**Table 2:** Diet composition of the weatherfish (*Misgurnus fossilis*) expressed as relative abundance (A%), frequency of occurrence (F%) of food categories and their relative importance index (IRI%) in two seasons and the dissimilarity in diets between seasons.

Food category	May			August			Dissimilarity		
	A%	F%	IRI%	A%	F%	IRI%	Average	Contribution %	Cumulative %
detritus	10.10	39.00	71.49	36.60	33.00	23.04	16.33	23.14	23.14
Chironomidae	20.00	51.00	100.00	29.20	31.00	25.17	11.28	15.99	39.13
Copepoda	24.20	55.00	112.51	0.07	20.00	8.08	10.84	15.36	54.49
*Asellus aquaticus*	18.10	52.00	96.80	6.43	16.00	8.00	8.28	11.73	66.22
Ephemeroptera	0.36	6.00	9.28	6.92	12.00	7.21	3.52	4.99	71.21
Coleoptera (larvae)	5.05	40.00	63.28	2.88	6.00	2.88	3.26	4.62	75.83
others	3.34	26.00	38.68	4.07	11.00	5.14	3.17	4.50	80.33
Oligochaeta	4.47	8.00	21.80	0.00	0.00	0.00	2.24	3.17	83.50
Chydoridae	3.67	47.00	71.71	0.07	3.00	1.10	1.82	2.58	86.08
Gastropoda	3.55	37.00	57.31	0.00	0.00	0.00	1.78	2.52	88.59
Ostracoda	3.46	51.00	77.28	0.51	13.00	4.90	1.63	2.31	90.91
Coleoptera (imago)	0.05	1.00	1.54	3.13	5.00	3.62	1.58	2.24	93.15
plants	1.47	29.00	42.83	1.29	5.00	2.10	1.20	1.17	94.86
Trichoptera	0.41	8.00	11.70	1.49	3.00	1.45	0.91	1.30	96.16
Zygoptera	0.06	1.00	1.44	1.75	3.00	1.43	0.90	1.28	97.43
Diptera others	0.74	3.00	4.91	0.79	3.00	1.19	0.74	1.05	98.49
Hirudinea	0.56	2.00	5.89	0.00	0.00	0.00	0.28	0.40	98.88
*Podura aquatica*	>0.01	1.00	1.42	0.54	4.00	1.53	0.27	0.38	99.27
Heteroptera	0.15	4.00	5.85	0.34	2.00	0.89	0.24	0.34	99.60
Cladocera different than Chydoridae	0.21	4.00	6.56	0.22	4.00	1.51	0.21	0.29	99.90
Hydracarina	0.05	10.00	14.30	0.10	2.00	0.74	0.07	0.10	100.00

The diet composition and importance of food items differed markedly between seasons (ANOSIM: R-statistic = 0.41, p < 0.001). SIMPER analysis showed that dissimilarity in the diet composition of fish sampled in May and August were attributable to detritus, chironomid larvae, *A. aquaticus* and copepods (Table 2). These four categories together constituted over 65.8% of cumulative dissimilarity in weatherfish diet between seasons.

Seasonal differences in diet composition corresponded with niche breadths (Table 3). All indices differed significantly between seasons but, on average, Levin’s (*B*) and Shannon-Wiener’s (*H*′) indices were 2–3 times larger in May than in August. Only their standardised forms; i.e. evenness indices (*B*_*a*_ and *J*′) showed smaller, but still significant, seasonal differences.

**Table 3:** Food niche indices of weatherfish (*Misgurnus fossilis*) and their differences in two seasons.

Index	May		August		Mann – Whitney test	
	Mean	SD	Mean	SD	z	p
Levins B	6.91	0.41	3.99	0.44	23.37	0.00001
Levins B_a_	0.29	0.02	0.17	0.02	13.46	0.00037
Shannon-Wiener H'	1.89	0.02	0.50	0.02	217.28	0.00001
Pielou J'	0.61	0.01	0.52	0.01	100.10	0.00001

The amount of detritus in the diet was not positively correlated with the abundance of other food categories (Table 4). Negative correlations were found in the quantity of different categories of benthic prey eaten; i.e. chironomid larvae and *A. aquaticus*, as well as typical planktonic groups; i.e. copepods and cladocerans (Table 4). These results imply that detritus was a source of food taken intentionally and independently from the other food items.

**Table 4:** Spearman’s rank correlation coefficients for the proportions of detritus with other food categories in the diet of weatherfish (*Misgurnus fossilis*).

Food category	May	August	Both
Chironomidae	0.273*	−0.449*	0.100
Copepoda	−0.215	−0.551*	−0.529*
*Asellus aquaticus*	−0.051	−0.416*	−0.383*
Ephemeroptera	0.003	−0.519*	−0.008
Coleoptera (larvae)	−0.434*	−0.086	−0.445*
others	−0.312*	−0.037	0.197
Oligochaeta	−0.182		−0.265*
Chydoridae	−0.354*	−0.368*	−0.596*
Gastropoda	−0.159		−0.422*
Ostracoda	0.222	−0.086	−0.217*
Coleoptera (imago)	0.126	−0.419*	0.015
plants	0.223	−0.322	−0.148
Trichoptera	−0.036	−0.128	−0.079
Zygoptera	−0.153	−0.007	0.064
Diptera others	0.112	−0.003	0.088
Hirudinea	−0.018		−0.092
*Podura aquatica*	−0.153	−0.025	0.063
Heteroptera	−0.007	−0.206	−0.060
Cladocera	−0.098	−0.504*	−0.187
Hydracarina	0.020	−0.346*	−0.187

*p < 0.05.

**Figure 3:**
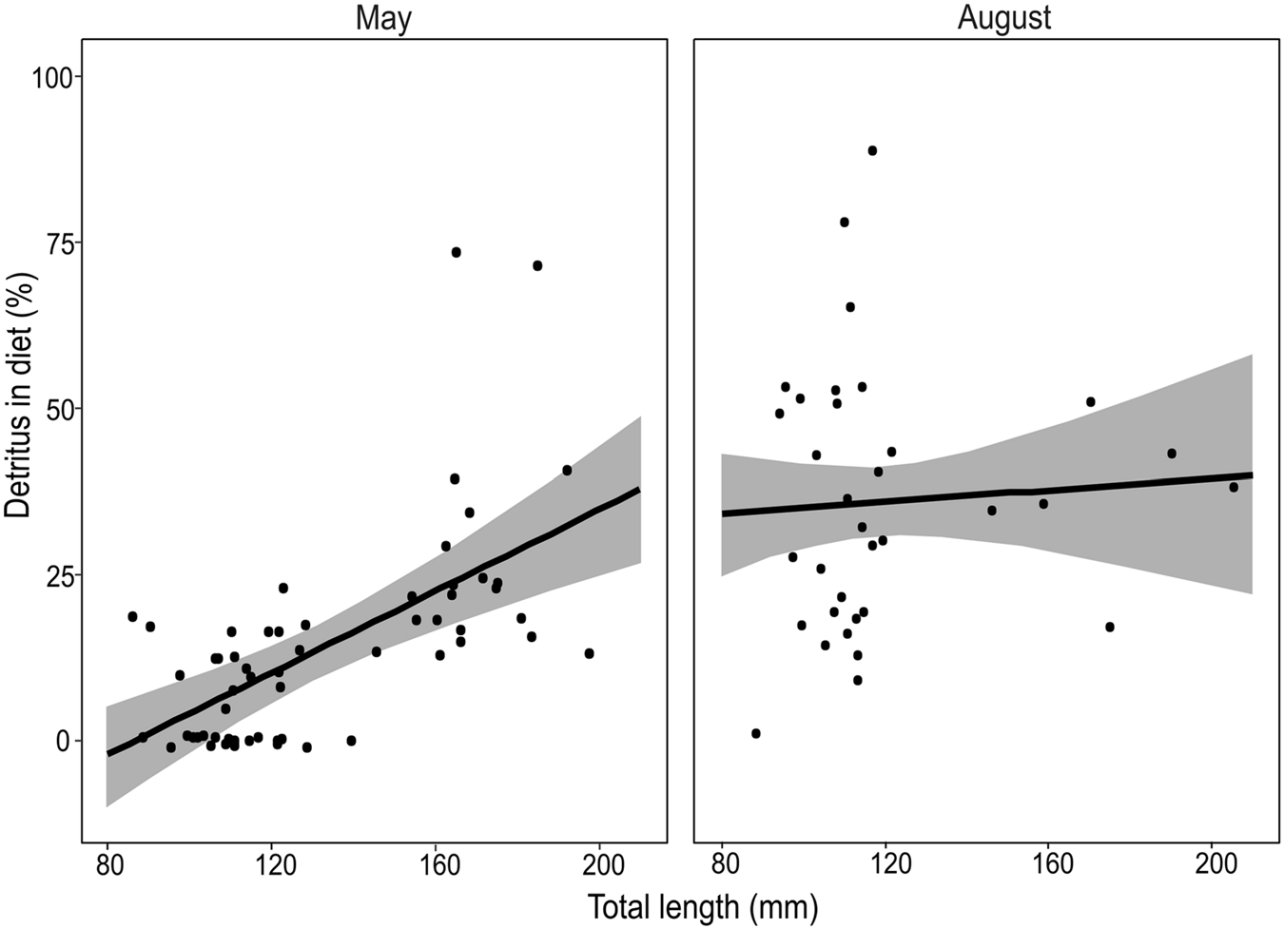
Mean fitted probability (solid line) and 95% confidence intervals (shaded area) of the proportion (%) of detritus in the diet of weatherfish (*Misgurnus fossilis*) against fish total length (mm) in two seasons (May and August).

The amount of detritus in the gut of weatherfish differed significantly between seasons (t = 5.674, df = 56, p < 0.001). In May the average proportion of detritus (arcsine transformed data) was lower than in August (13.70 ± 3.70 and 36.14 ± 6.01, respectively). The amount of detritus consumed by weatherfish showed strong temporal dependency, varying with season, and also as a function of fish size, at least in May (Fig. 3). Notably, the GLM model showed a significant interaction between fish TL and season in the proportion of detritus consumed (Table 5). Thus, while there was a strong positive relationship between TL and detritus consumption in May, this was not the case in August (Fig. 3).

**Table 5:** Summary of Gaussian GLM model of the proportion (%) of detritus in the diet of weatherfish (*Misgurnus fossilis*) as a function of total length (mm) in two seasons (May and August).

Model parameter	Estimate	SE	p
Intercept_(May)_	−26.65	8.70	<0.001
Total length	3.07	0.64	<0.001
Time_(August)_	57.56	14.67	<0.001
Total length × Time_(August)_	−2.63	1.16	0.026

In weatherfish the relationship between alimentary tract length (AtL) and fish size (TL) was linear and took the form: AtL = 0.499 (0.011) × TL − 3.919 (1.345); r_a_^2^ = 0.950, n = 120, p < 0.001. A power relationship gave a slightly poorer fit to the data (r_a_^2^ = 0.937), but the estimated slope (±s.e.) (1.058 ± 0.025) indicated that the relationship was slightly positively allometric; i.e. b > 1 (t = 2.318, df = 118, p = 0.022). There was no difference in the slopes of the regression of alimentary tracts length on total length between seasons (F_1,118_ = 2.404; p = 0.077).

## Discussion

Our results demonstrated a distinct diel pattern to weatherfish feeding activity. Among loach species, diel feeding activity has been demonstrated in stone loach (*Barbatula barbatula*)^38^, spined loach (*Cobitis taenia*)^39^ and golden loach (*Sabanejewia aurata*)^40^. All species show nocturnal activity with a peak of feeding during the night. In contrast, weatherfish appear to feed actively throughout the day with the highest feeding activity during daylight hours. Under relatively benign oxygen conditions at the study site during May, the peak of fish feeding activity occurred during the period of the greatest light intensity and highest dissolved oxygen concentration. In August, weatherfish showed similar feeding activity throughout the day, but with a small increase in feeding activity during the night, coinciding with a decrease in water temperature. Fish diel activity can be plastic, changing with endogenous circadian mechanisms as well as environmental factors, such as light intensity, temperature or season^41^. Nocturnal feeding in stream fish is generally considered as predator avoidance behavior^42^. A reduction in feeding activity can be caused by a scarcity of food and the impact of unfavourable oxygen and temperature conditions, which are the main factors that affect feeding^43^. In August, when the water surface at the study site was covered by dense vegetation, light penetration was limited and dissolved oxygen concentration reduced and a diel feeding pattern was not observed. Kostromarova^44^ reported that the optimum temperature for the development of *M. fossilis* larvae is 18.0–21.5 °C, and a temperature above 24 °C is considered to be lethal during the embryonic period^45^. In August, we recorded a maximum water temperature exceeding 23.5 °C, which may influence fish activity including feeding behaviour.

Published data on the diet and feeding pattern of weatherfish are scarce. In general, as a bottom-dwelling fish with small eyes and mouth, *M. fossilis* feed mainly on small benthic invertebrate as well as larvae of dipterans, crustaceans or molluscs, selecting prey by tactile and chemical cues using oral barbels^46,47^. At the study site, weatherfish fed on a large spectrum of food categories, though the diet was dominated by larvae of macroinvertebrates (Chironomidae, Coleoptera, Ephemeroptera), zooplankton and detritus. The diet composition was similar to related species, such as the oriental weatherfish (*Misgurnus anguillicaudatus*), which also feeds mainly on small benthic invertebrates, such as mayflies, caddisflies, chironomid larvae^48^, small amount of detritus and plant debris^49,50^, as well as on zooplankton^51^. Frable^52^ also showed that oriental weatherfish are primarily omnivores and feed on benthic invertebrates (insect larvae, snails, worms, ostracods, cladocerans), fish eggs, algae and detritus.

The large diversity of prey we recorded in the diet in May showed that weatherfish can be viewed as a typical opportunistic feeder, using the most readily available food sources. Thus, insect larvae associated with aquatic vegetation (Ephemeroptera, Coleoptera) were found in the diet as well as a significant amount of zooplankton (Copepoda and Cladocera, primarily Chydoridae). Plant items in the diet mostly comprised duckweed (*Lemna* sp.) and seeds, while animals of terrestrial origin were also recorded (classified in the category ‘others’). Plant material in the diet may be ingested accidentally with other food items, and potentially also when gulping air at the water surface^14^. The broad feeding niche exhibited by weatherfish may result from a lack of competitors. At the study site and in published studies, weatherfish usually occur alone or with other fish species present only occasionally^16,23^.

The diet of the weatherfish was also shown to vary seasonally. Differences in feeding conditions between May and August were mirrored by the proportion of fish with empty guts. In total, 22.5% of weatherfish had empty guts and specimens with empty alimentary tracts were observed primarily in August. In May, when better conditions for feeding occurred, the fullness coefficient was higher and the diet composition in terms of the amount of food, number and diversity of prey was significantly different from that recorded in late summer. This difference in gut fullness between seasons may reflect a decline in feeding rate in late summer, a more rapid rate of processing of food items by the gut at elevated temperatures, or both. In August, detritus and chironomids, especially *Chironomus* sp.; a taxon known to be resistant to low dissolved oxygen conditions^53^, were the main food categories. Moreover, detritus was the primary food item contributing to the dissimilarity in diet composition between seasons. Diet switching from higher- to lower-energy sources as food availability declines is a common strategy used by omnivorous fish to withstand harsh periods^54,55^. In unfavourable environmental conditions, with restricted food resources, high water temperature and low dissolved oxygen concentration, like that seen in August, detritus was the main food resources for the full size spectrum of individuals. In May, however, detritivory was size-dependent, with only the largest individuals consuming detritus.

Detritus may occur in the diet of weatherfish as an unintentional by-product of substrate feeding^56^ or from the digestion of detritivorous prey (e.g. Chironomidae larvae). However, the marked increase in the amount of detritus in the diet observed in August implies its importance as a food item at this time (Table 2).Detritus may represent a critical source of nutrients and biogenous elements, such as nitrogen and carbon^57^. A study conducted by Urquhart and Koetsier^56^ on the oriental weatherfish showed that the main component of the diet was macroinvertebrates, and in particular chironomid larvae, which is typical of benthic freshwater fish. Our results indicate that detritus is not only an important food category for weatherfish, but in the absence of other available prey may be the main component of its diet. Detritivory is a common feeding tactic mainly among tropical fish^58,59^. In European fishes, a diet of detritus is relatively rare and few species are recognised as detritivorous, examples include European bitterling (*Rhodeus amarus*)^60^ and ide (*Leuciscus idus*)^61^. Other fish can switch to detritus temporarily when preferred foods are not available^59^. Detritivorous fishes show specific anatomical and/or physiological adaptations for the collection and digestion of detritus^62^. One of the important adaptations is the length of alimentary tract and fish that are able to utilize detritus tend to have an extremely long and coiled intestine, often more than five times the length of the fish^60,63,64^ and a long intestine and absence of a well-defined stomach is a characteristic of cyprinids and other bottom-feeding fishes that consume large quantities of detritus^65^. Notably, plasticity in the length of the digestive tracts of species that experience temporal or spatial differences in food quality is recognised^66–68^. Unusually, although weatherfish do not have a distinct stomach, their intestine is short and straight and can be divided into two parts: the anterior which is glandular and morphologically suited to digestion and the posterior, which has the form of a straight tube. Both parts are separated by spiral zone which compacts the undigested material to keep the gut wall free to facilitate gas exchange. The short gastrointestinal tract may reflect the fact that weatherfish are primarily carnivorous, feeding on macroinvertebrates^69^. Thus, weatherfish detritivory may represent a suboptimal and temporary feeding tactic that represents an adaptive response to unfavourable conditions^64^ that is not reflected by morphological adaptations to the alimentary tract.

## Conclusion

The wide range of prey utilised by weatherfish, reflecting the temporal dynamics of available food resources in a highly altered habitat, indicate an opportunistic feeding strategy. This mode of feeding may contribute to the success of the highly endangered weatherfish at the study site, which was otherwise almost fishless. It is notable that in the presence of competitors and predators this species is never abundant. The capacity of weatherfish to establish and maintain robust populations in ostensibly sub-optimal habitats for fish, may reflect its ability to utilise abundant but low-quality food items, such as detritus.
